# Effectiveness of ixekizumab for the treatment of moderate‐to‐severe plaque psoriasis with involvement of difficult‐to‐treat areas: A 52‐week multicenter retrospective study

**DOI:** 10.1111/1346-8138.17115

**Published:** 2024-01-31

**Authors:** Mario Valenti, Luigi Gargiulo, Luciano Ibba, Andrea Cortese, Francesco Toso, Diego Orsini, Viviana Lora, Pasquale Frascione, Paolo Sena, Andrea Carugno, Chiara Assorgi, Antonio Costanzo, Alessandra Narcisi

**Affiliations:** ^1^ Dermatology Unit IRCCS Humanitas Research Hospital Rozzano Italy; ^2^ Department of Biomedical Sciences Humanitas University Pieve Emanuele Italy; ^3^ Clinical Dermatology Unit San Gallicano Dermatological Institute IRCCS Rome Italy; ^4^ Dermatology Oncology Unit San Gallicano Dermatological Institute IRCCS Rome Italy; ^5^ Dermatology Unit ASST Papa Giovanni XXIII Bergamo Italy; ^6^ Ph.D. Program in Molecular and Translational Medicine (DIMET) University of Milan‐Bicocca Milan Italy; ^7^ Section of Dermatology, Department of Clinical Medicine and Surgery University of Naples Federico II Napoli Italy

**Keywords:** biologics, ixekizumab, psoriasis, psoriasis treatment, real‐world

## Abstract

Biological drugs have dramatically changed the approach to treating moderate‐to‐severe plaque psoriasis, achieving excellent skin clearance and safety outcomes. However, the management of difficult‐to‐treat areas (e.g., scalp, palms/soles, nails, and genitalia) still represents a challenge in psoriasis treatment. Data in the literature on difficult‐to‐treat sites are limited and, frequently, no specific analysis is performed during clinical trials. We conducted a 52‐week, retrospective study to evaluate the effectiveness of ixekizumab in 120 patients with moderate‐to‐severe plaque psoriasis of at least one difficult‐to‐treat area (scalp, palmoplantar surfaces, nails, and genitalia). Ninety‐nine patients had scalp psoriasis, 35 had involvement of the palms or soles, 27 were affected by genital psoriasis, and 22 patients reported involvement of the nails. After 1 year of treatment, 96% of patients with scalp involvement, 95.6% of patients with palmoplantar psoriasis, 95.2% of patients with genital psoriasis, and 85% of patients with nail involvement achieved a site‐specific Physician's Global Assessment of 0 or 1 (clear or almost clear). No serious adverse events were observed during the study. Our study supports the effectiveness of ixekizumab in plaque psoriasis involving difficult‐to‐treat sites.

## 1 INTRODUCTION

Plaque psoriasis is a chronic inflammatory disease involving the entire cutaneous surface, most commonly affecting areas such as the elbows or knees. However, difficult‐to‐treat areas, such as the scalp, palms/soles, nails, and genitalia, are frequently involved.^1^

The treatment of these surfaces is often challenging due to the low compliance to topical regimens and the primary/secondary ineffectiveness of conventional systemic drugs such as cyclosporin, methotrexate, or acitretin.^2^ Furthermore, long‐term exposure to systemic conventional drugs can lead to safety issues such as liver or kidney function impairment, blood count abnormalities, and severe mucocutaneous xerosis.^2^

Biological drugs have changed the treatment paradigm of moderate‐to‐severe plaque psoriasis.^3^ However, only limited data are available regarding their use in patients with a lower body surface area (BSA) affected and the predominant involvement of difficult‐to‐treat areas.^4^, ^5^ This is primarily due to the strict inclusion criteria of clinical trials, which usually enroll patients with a Psoriasis Area and Severity Index (PASI) and/or a BSA score of at least 10. Among the biological drugs approved for plaque psoriasis, ixekizumab is an inhibitor of interleukin (IL)‐17A that has been evaluated in several clinical trials demonstrating effectiveness in difficult‐to‐treat areas. This multicenter study aimed to assess the effectiveness and safety of ixekizumab in patients affected by plaque psoriasis involving difficult sites.

## 2 METHODS

We enrolled 120 adult patients affected by plaque psoriasis with moderate‐to‐severe involvement of at least one difficult‐to‐treat area (defined by a site‐specific Physician's Global Assessment [PGA] ≥ 3), all treated with ixekizumab. Patients' demographic characteristics were retrospectively retrieved from the database of three Italian dermatology units. Ixekizumab was administered according to the Summary of Product Characteristics.^6^ Scalp‐specific PGA (sc‐PGA), palmoplantar PGA (ppPGA), static PGA of the genitalia (sPGA‐G), fingernail PGA (f‐PGA), and PASI were recorded at each dermatological examination. For each area, the severity of psoriasis was evaluated clinically on a scale from 0 (absence of lesions) to 4 (severe involvement of the area). The possible occurrence of any adverse events (AEs) was also evaluated throughout the study period. The primary endpoints were the proportion of patients achieving a site‐specific PGA of 0/1 (clear or almost clear) at weeks 16, 28, and 52. Continuous data were reported as mean and standard deviation (SD), while categorical variables were presented as absolute numbers and percentages. The within‐group comparison of mean PGA (between baseline and weeks 16, 28, and 52) was performed using the Student's *t‐*test.

The procedures for this study adhered to clinical standards and did not necessitate approval from the institutional review board. All patients provided written consent for their anonymous data to be retrieved retrospectively. The research complied with the Helsinki Declaration of 1964 and its later amendments.

## 3 RESULTS

**FIGURE 1:**
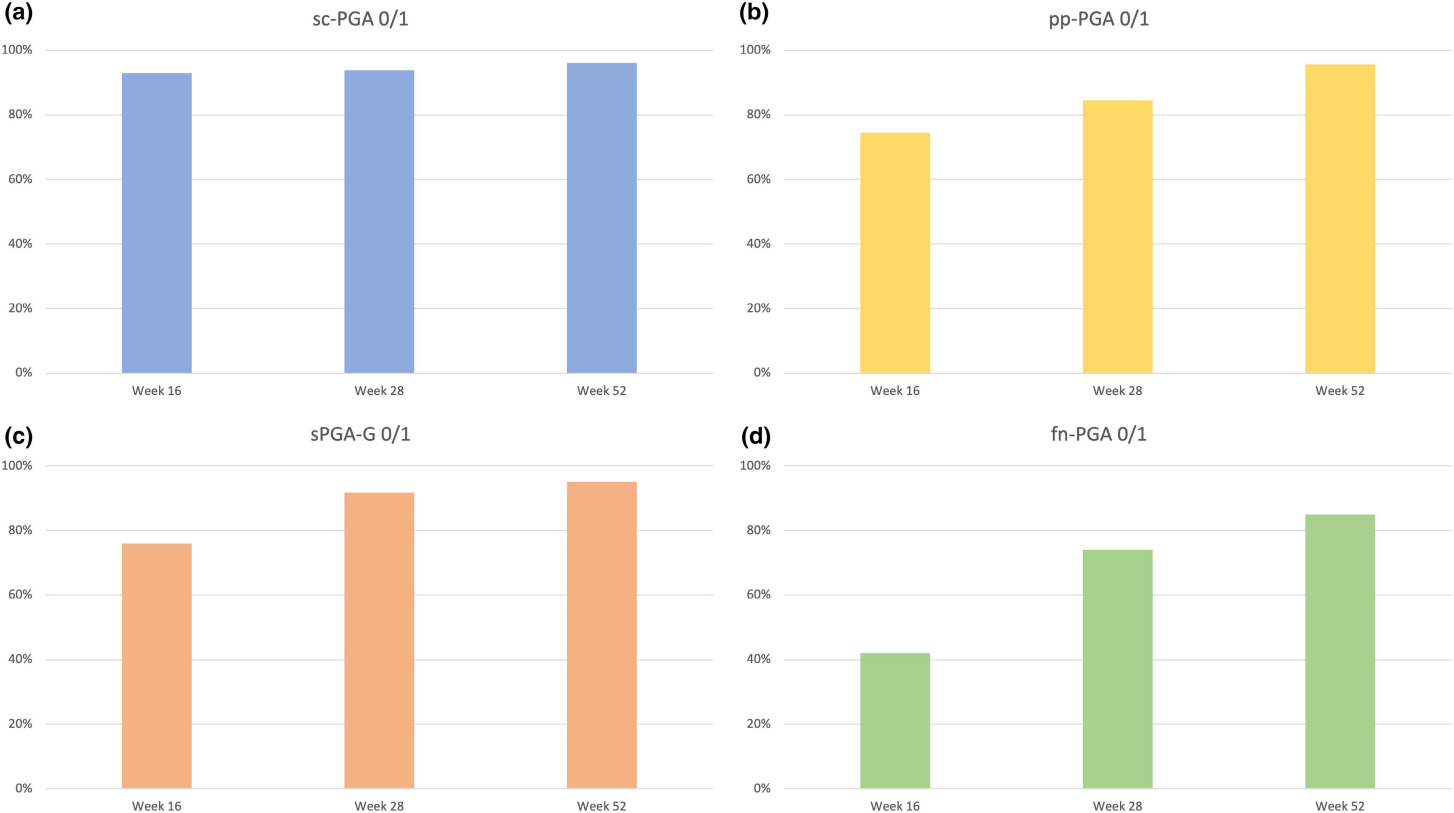
Percentages of patients achieving a site‐specific Physician's Global Assessment (PGA) score of 0/1 (clear or almost clear) throughout the study period regarding involvement of (a) the scalp (sc‐PGA), (b) palmar and/or plantar regions (pp‐PGA), (c) genitalia (s‐PGA‐g), and (d) fingernails.

**FIGURE 2:**
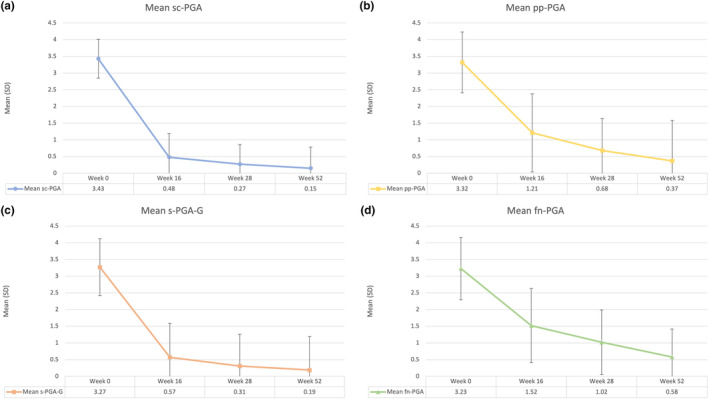
Reduction of mean (standard deviation) site‐specific Physician's Global Assessment (PGA) during 52 weeks concerning each difficult‐to‐treat area. (a) scalp (sc‐PGA), (b) palmar and/or plantar regions (pp‐PGA), (c) genitalia (s‐PGA‐g), and (d) fingernails (fn‐PGA).

We included 120 patients treated with ixekizumab for at least 52 weeks (Table 1). Seventy‐nine were males (65.8%) with a mean (SD) age of 49.01 (15.42) years. Our patients were affected by psoriasis for a mean of 19.01 (15.45) years. The mean body mass index (BMI) was 29.16 (6.40), while 32 patients (26.7%) had at least one cardiometabolic comorbidity (arterial hypertension, obesity, type II diabetes mellitus, hypercholesterolemia, and cardiovascular diseases). Twenty‐two patients had a concomitant diagnosis of psoriatic arthritis (18.3%). Twenty‐nine patients had previously received at least one biological drug (24.2%). Regarding the 99 patients with scalp involvement, an sc‐PGA of 0/1 was achieved by 92.9%, 93.9%, and 96% of them at weeks 16, 28, and 52, respectively (Figure 1a). After 16 weeks, 74.5% of the 37 patients with palmoplantar psoriasis showed a ppPGA of 0/1 with continuous improvement at weeks 28 (84.5%) and 52 (95.6%) (Figure 1b). Among the 26 patients with genital psoriasis, an sPGA‐G of 0/1 was observed in 76% at week 16, 91.7% at week 28, and 95.2% after 1 year of treatment (Figure 1c). Finally, for the 24 patients with nail involvement, an f‐PGA of clear or almost clear was reached by 42% at week 16, 74% at week 28, and 85% at week 52 (Figure 1d). Mean site‐specific PGA improved in each difficult‐to‐treat area during the study. At baseline, the mean sc‐PGA was 3.43 and decreased to 0.48 at week 16, 0.27 at week 28, and 0.15 after 1 year of treatment (Figure 2a). Mean pp‐PGA decreased from a mean of 3.32 at baseline to 1.21 at week 16, 0.68 at week 28, and 0.37 at week 52 (Figure 2b). Mean sPGA‐G decreased from a mean of 3.27 at baseline to 0.57, 0.31, and 0.19 at 16, 28 and 52 weeks (Figure 2c). Mean f‐PGA was 3.23 at baseline and decreased to 1.52 at week 16, 1.02 at week 28, and 0.58 after 52 weeks of treatment (Figure 2d). At baseline, the mean (SD) PASI of our cohort was 12.48 (7.81). During the study, it decreased to 1.53 (1.80) at week 16, 0.58 (1.45) at week 28, and 0.38 (1.39) at week 52.

**TABLE 1:** Demographic and clinical characteristics of the study population at baseline.

Number of patients, *N*	120
Males, *n* (%)	79 (65.8)
Age, mean ± SD	49.01 ± 15.42
BMI, mean ± SD	29.16 ± 6.40
Disease duration, mean ± SD	19.01 ± 15.45
PsA, *n* (%)	22 (18.3)
Cardiometabolic comorbidities	32 (26.7)
Bio‐experienced, *n* (%)	29 (24.2)
PASI baseline, mean ± SD	12.48 ± 7.81

Abbreviations: BMI, body mass index; PASI, Psoriasis Area and Severity Index; PsA, Psoriatic arthritis; SD, standard deviation.

No significant safety findings were reported during the study, as no patient had to discontinue the drug because of treatment‐emergent AEs or serious AEs.

## 4 DISCUSSION

Phase‐3 clinical trials have demonstrated that ixekizumab is an efficacious option for the treatment of difficult sites. In our real‐world experience, we observed similar responses compared with those from clinical trials. Regarding scalp psoriasis, an analysis of the UNCOVER‐1, UNCOVER‐2, UNCOVER‐3 studies showed a reduction of 90% and 100% on the Psoriasis Scalp Severity Index in 75.6% and 65.9% of patients respectively at week 12.^7^ Narcisi et al.,^4^ observed an s‐PGA of 0 or 1 in 93.1% and 91.6% of patients treated with ixekizumab after 16 and 24 weeks, respectively.

A subpopulation analysis of the UNCOVER studies also supports the use of ixekizumab in non‐pustular palmoplantar psoriasis as approximately half of the patients achieved complete skin clearance of the palms and soles after 12 weeks of treatment.^8^

A specific clinical trial was conducted to evaluate the role of ixekizumab in the treatment of genital psoriasis (IXORA‐Q).^9^ In this study, clear or almost clear genital skin was achieved by 73% of patients at week 12 and 75% after 1 year of treatment. A site‐specific PGA of 0 was observed in 56% of patients after 12 weeks.

Regarding nail psoriasis, the IXORA‐S clinical trial, compared the efficacy of ixekizumab with ustekinumab after 52 weeks in terms of the Nail Psoriasis Severity Index (NAPSI).^10^ In particular, a NAPSI score of 0 was reached by 61.9% of patients with a mean decrease from baseline of 22.4 points after 52 weeks of treatment with ixekizumab. In comparison, patients treated with ustekinumab in this study experienced a mean NAPSI decrease of 15.6 points from baseline with just 28.6% of them achieving complete nail clearance.^10^ These findings were consistent with those from a subpopulation analysis of the UNCOVER‐3 clinical trial, which reported a NAPSI of 0 in more than half of patients after 60 weeks of treatment with ixekizumab.^11^ In addition, a recent network meta‐analysis showed that ixekizumab had the greatest likelihood among approved biologics of achieving complete resolution of nail psoriasis at week 24.^12^ Among interleukin inhibitors, ixekizumab and risankizumab appear to be the drugs with the highest effectiveness on difficult‐to‐treat areas, according to data from both clinical trials and real‐world experience.^9^, ^10^, ^11^, ^12^, ^13^

The safety profile of ixekizumab in our study was very high as no serious AEs or AEs leading to discontinuation were observed, consistent with the results of a 5‐year analysis of the UNCOVER‐3 study.^14^

The main limitations of this study include are the retrospective nature and the absence of a control group, which do not allow the generalization of our findings.

Our experience supports data from clinical trials and meta‐analyses regarding the high efficacy of ixekizumab in plaque psoriasis with the predominant involvement of difficult‐to‐treat areas. Therefore, ixekizumab could be considered as a first‐line biological drug when treating this subset of patients. However, larger and longer studies are needed to further investigate the effectiveness and safety of ixekizumab in psoriasis patients with moderate‐to‐severe involvement of difficult sites.

## CONFLICT OF INTEREST STATEMENT

Mario Valenti has been a consultant and/or speaker for Sanofi, Leo Pharma, Eli Lilly, Novartis, Janssen, AbbVie, and Boehringer Ingelheim. Luigi Gargiulo has been a consultant for Almirall. Antonio Costanzo has served as an advisory board member, consultant, and has received fees and speaker's honoraria or has participated in clinical trials for Abbvie, Almirall, Biogen, LEO Pharma, Lilly, Janssen, Novartis, Pfizer, Sanofi Genzyme, and UCB‐Pharma. Alessandra Narcisi has served on advisory boards, received honoraria for lectures and research grants from Almirall, Abbvie, Leo Pharma, Celgene, Eli Lilly, Janssen, Novartis, Sanofi‐Genzyme, Amgen and Boehringer Ingelheim. The other authors have no conflicts of interest to disclose.

## ETHICAL STATEMENT

Institutional review board approval was exempted as the study protocol did not deviate from standard clinical practice. The patients received ixekizumab as in good clinical practice in accordance with European guidelines. The patients provided written consent for retrospective study of data collected during routine clinical practice (demographics, clinical scores). The study was performed in accordance with the Helsinki Declaration of 1964 and its later amendments. Data collection and handling complied with applicable laws, regulations, and guidance regarding patient protection, including patient privacy.

## Data Availability

Data available on request from the authors.
